# Experimental and Clinical Evidence Suggests That Treatment with Betacellulin Can Alleviate Th2-Type Cytokine-Mediated Impairment of Skin Barrier Function

**DOI:** 10.3390/ijms231911520

**Published:** 2022-09-29

**Authors:** Ge Peng, Saya Tsukamoto, Yoshie Umehara, Ryoma Kishi, Mitsutoshi Tominaga, Kenji Takamori, Ko Okumura, Hideoki Ogawa, Shigaku Ikeda, François Niyonsaba

**Affiliations:** 1 Department of Dermatology and Allergology, Juntendo University Graduate School of Medicine, Tokyo 1138421, Japan; 2 Atopy (Allergy) Research Center, Juntendo University Graduate School of Medicine, Tokyo 1138421, Japan; 3 Juntendo Itch Research Center (JIRC), Institute for Environmental and Gender-Specific Medicine, Juntendo University Graduate School of Medicine, Chiba 2790021, Japan; 4 Department of Dermatology, Juntendo University Urayasu Hospital, Chiba 2790021, Japan; 5 Faculty of International Liberal Arts, Juntendo University, Tokyo 1138421, Japan

**Keywords:** betacellulin, atopic dermatitis, biomarker, keratinocyte, skin barrier

## Abstract

Betacellulin (BTC) is a peptide ligand that belongs to the epidermal growth factor family, the members of which have been implicated in skin morphogenesis, homeostasis, repair, and angiogenesis; however, the role of BTC in the regulation of the skin barrier remains unknown. To examine the role of BTC in skin barrier function, we analyzed atopic dermatitis (AD) transcriptomic data from Gene Expression Omnibus (GEO) datasets, performed BTC immunohistochemistry using human skin tissues, and evaluated the effects of BTC on primary human keratinocytes by real-time PCR, Western blotting, and assay of the transepidermal electrical resistance (TER), a functional parameter to monitor the tight junction barrier. We found that the gene expression of BTC was downregulated in skin lesions from patients with AD, and this downregulated expression recovered following biological treatments. Consistently, the BTC protein levels were downregulated in the lesional skin of AD patients compared with the normal skin of healthy participants, suggesting that the BTC levels in skin might be a biomarker for the diagnosis and therapy of AD. Furthermore, in human keratinocytes, BTC knockdown reduced the levels of skin-derived antimicrobial peptides and skin barrier-related genes, whereas BTC addition enhanced their levels. Importantly, in human skin equivalents, BTC restored the increased tight junction permeability induced by Th2 cytokine IL-4/IL-13 treatment. In addition, specific inhibitors of epidermal growth factor receptor (EGFR) and protein kinase C (PKC) abolished the BTC-mediated improvement in skin barrier-related proteins in keratinocyte monolayers. Collectively, our findings suggest that treatment with BTC might improve the Th2-type cytokine-mediated impairment of skin barrier function through the EGFR/PKC axis and that BTC might be a novel potential biomarker and therapeutic target for the treatment of skin conditions characterized by the overproduction of Th2 cytokines and dysfunctional skin barriers, such as AD.

## 1. Introduction

Atopic dermatitis (AD) is a chronic inflammatory skin condition characterized by fluctuating symptoms, including pruritus and eczematous lesions [1]. AD is the most common skin condition worldwide and affects over 200 million people. Up to one-third of AD cases are considered moderate to severe. Although AD has long been associated with skin barrier dysfunction, the pathogenesis of this condition is more complex and involves various molecular markers in different functional domains. The objective of AD treatment is the induction and long-term maintenance of a remission state in which no symptoms appear or symptoms are mild and do not interfere with daily life. For the induction and maintenance of remission, in addition to relying on the application of topical steroids, which is not sufficient for the treatment of intractable AD, researchers have been increasingly focusing on targeting pathogenic factors, including skin dysbiosis, Th2-skewed inflammation, the impairment of both the stratum corneum (SC) barrier and the tight junction (TJ) barrier, and the breakage of skin-derived antimicrobial peptides [2].

Betacellulin (BTC) is a peptide growth factor that directly binds to and activates epidermal growth factor receptor (EGFR) [3]. Abundant evidence shows that the normal development and homeostasis of the skin depend on the correct expression and activity of EGFR and its ligands [4,5]. Overexpression of EGFR has been detected in psoriasis [6], and increased levels of multiple EGFR ligands, such as epiregulin, are found in the psoriatic epidermis [7]. Although BTC is expressed in the skin, its roles in the maintenance of the skin barrier and the regulation of inflammation are largely unknown. In human skin, BTC expression appears to be restricted to suprabasal keratinocytes, particularly to the granular cell layer [8], and its expression is downregulated in psoriatic skin [8,9]. In addition, BTC plays a protective role against inflammation in pancreatitis [10]. Noticeably, *Staphylococcus aureus* (*S. aureus*) skin colonization is frequent in AD and common in cancer patients treated with EGFR inhibitors [11]. In addition, skin dysbiosis is partially dependent on impaired EGFR signaling [11], whereas EGF relieves inflammatory signals in *S. aureus*-treated human epidermal keratinocytes and AD-like skin lesions in NC/Nga mice [12]. Moreover, a deficiency of SC barrier proteins, such as filaggrin and loricrin, leads to AD-like inflammation and a reduction in the EGFR level [13]. Similarly, EGFR signaling attenuates the development and relapse of AD in a murine model [12]. However, the roles of BTC, a ligand of EGFR, in skin inflammation and skin barrier function in AD remain unclear.

Therefore, in the present study, the clinical significance and diagnostic value of BTC in patients with AD were evaluated by analyzing data from the Gene Expression Omnibus (GEO) database. In addition, the distribution of BTC in skin tissues from AD patients and healthy subjects and the effects of the silencing and extracellular addition of BTC on normal human keratinocytes were analyzed. Furthermore, we investigated the role of and mechanism through which BTC contributes to the regulation of skin barrier function by using human skin equivalents and human primary keratinocytes.

## 2. Results

### 2.1. The Gene Expression of BTC Is Downregulated in the Lesional Skin of AD Patients

**Figure 1:**
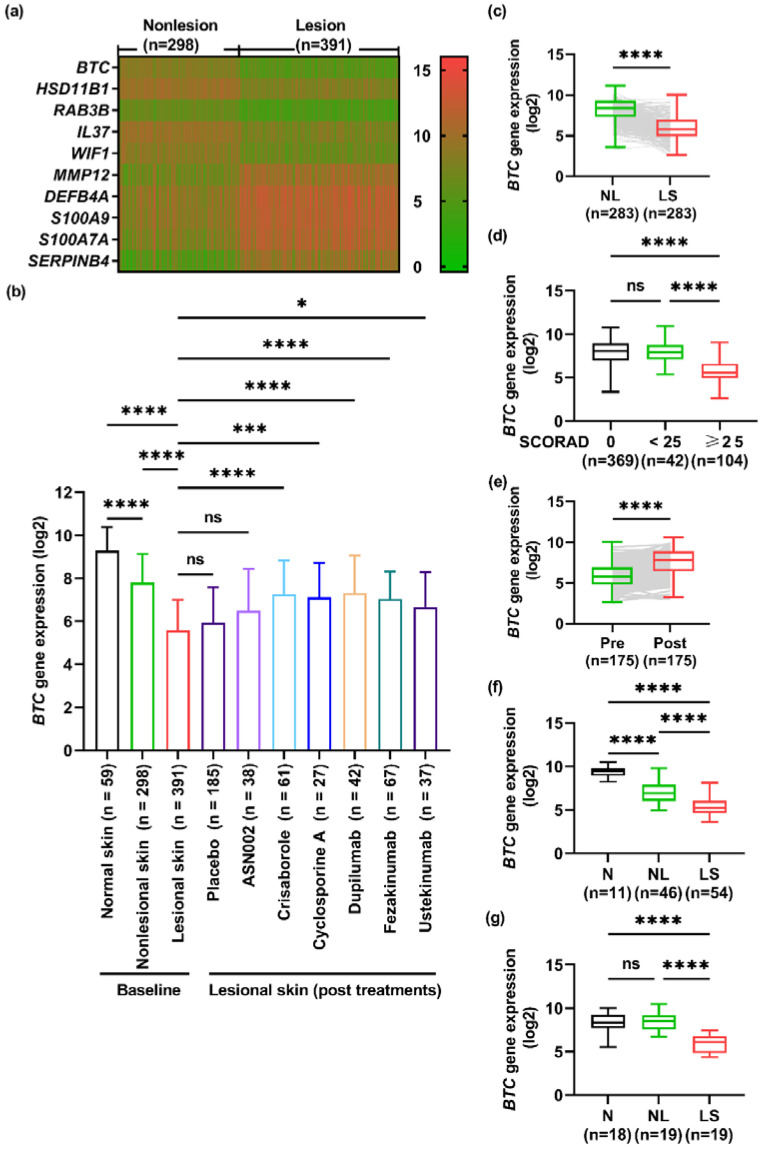
The gene expression of *BTC* is downregulated in the lesional skin of AD patients. (**a**). Heatmap of the top 10 differentially expressed genes between nonlesional and lesional human AD skin tissues. (**b**). The mRNA level of *BTC* was markedly decreased in the skin lesions of patients with AD (*n* = 391) compared with nonlesional AD skin tissues (*n* = 298) and normal skin tissues (*n* = 59) and recovered after treatment with crisaborole (*n* = 61), cyclosporine A (*n* = 27), dupilumab (*n* = 42), fezakinumab (*n* = 67) or ustekinumab (*n* = 37) but not ASN002 (*n* = 38) or placebo (*n* = 185). (**c**). The mRNA level of *BTC* was also decreased in the skin lesion tissues compared with paired nonlesional skin tissues (*n* = 283). (**d**). The mRNA level of *BTC* was noticeably decreased in the skin lesional tissues of patients with AD with a SCORAD over 25 (*n* = 104) compared with skin lesions with a SCORAD lower than 25 (*n* = 42) or equal to 0 (*n* = 369). (**e**). The mRNA level of *BTC* in skin lesions of patients with AD after the treatment recovered to the levels found in paired skin lesional tissues before the treatment (*n* = 283). (**f**). The mRNA level of *BTC* was markedly decreased in the skin lesions of adult patients with AD (*n* = 54) compared with nonlesional (*n* = 46) and normal tissues (*n* = 11). (**g**). The mRNA level of *BTC* was also decreased in the skin lesions of children with AD (*n* = 18) compared with nonlesional (*n* = 19) and normal tissues (*n* = 19). ns > 0.05 (non significant), * *p* < 0.05, *** *p* < 0.001 and **** *p* < 0.0001.

We collected samples from the GEO database, including samples of normal skin from healthy donors and samples of nonlesional skin and lesional skin from patients with AD. Figure 1a displays the top 10 differentially expressed genes between nonlesional and lesional AD skin tissues, and among these, *BTC* exhibited the greatest downregulation. Compared with the levels in nonlesional skin tissues (*n* = 298) and normal skin tissues (*n* = 59), the gene expression level of *BTC* was markedly decreased in lesional skin tissues from patients with AD (*n* = 391) (Figure 1b). Moreover, significantly reduced *BTC* gene levels could also be observed in AD lesional skin compared with nonlesional skin (*n* = 283) (Figure 1c). Furthermore, a scoring index, SCORing Atopic Dermatitis (SCORAD), was used to separate the AD patients into mild (SCORAD < 25) and moderate to severe (SCORAD ≥ 25) groups, whereas the SCORAD of normal skin was set to zero. Obviously, the *BTC* gene level in the moderate to severe group was significantly decreased compared with that in the mild and normal groups (Figure 1d). By comparing the expression level of *BTC* in AD lesional skin before and after biological treatments with crisaborole (phosphodiesterase inhibitor), dupilumab (IL-4/IL-13 receptor monoclonal antibody), fezakinumab (IL-22 monoclonal antibody), or ustekinumab (anti-IL-12/IL-23 antibody) or systematic treatment with cyclosporine A but not ASN002 (oral dual inhibitor of Janus kinase and spleen tyrosine kinase) or placebo, we found that the *BTC* level recovered after the treatments, and this recovery was accompanied by the relief of skin inflammation (Figure 1b,e). Importantly, a reduction in the *BTC* levels in lesional skin was found in both adults (Figure 1f) and children (Figure 1g).

### 2.2. The Diagnostic Value of BTC in AD Patients

**Figure 2:**
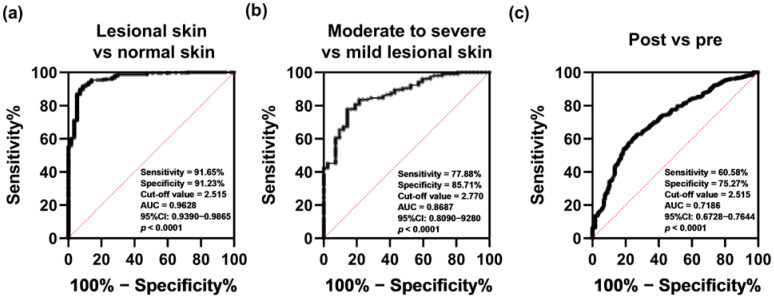
Diagnostic value of BTC in AD. The expression level of BTC in AD can be used to distinguish skin lesions from normal skin (**a**), moderate to severe skin lesions from mild skin lesions (**b**), and pretreatment lesions from posttreatment lesions (**c**).

We subsequently investigated the diagnostic value of BTC in AD by receiver operating characteristic (ROC) curve analysis. The optimal cutoff value to distinguish patients with AD from healthy individuals was BTC < 2.515, with an area under the ROC curve (AUC) of 0.9628, a sensitivity of 91.65% and a specificity of 91.23% (Figure 2a). Similarly, the BTC level (<2.770) was sufficient to distinguish moderate to severe AD lesional skin from mild AD lesional skin, with an AUC of 0.8687, a sensitivity of 77.88% and a specificity of 85.71% (Figure 2b). Unexpectedly, a BTC cutoff value less than 2.515 could appropriately distinguish pretreatment lesions from posttreatment lesions in patients with AD with a good response to biological treatments, including crisaborole, dupilumab, fezakinumab, and ustekinumab (Figure 2c).

### 2.3. Cutaneous Expression and Distribution of BTC and Effects of Its Knockdown on Human Keratinocytes

**Figure 3:**
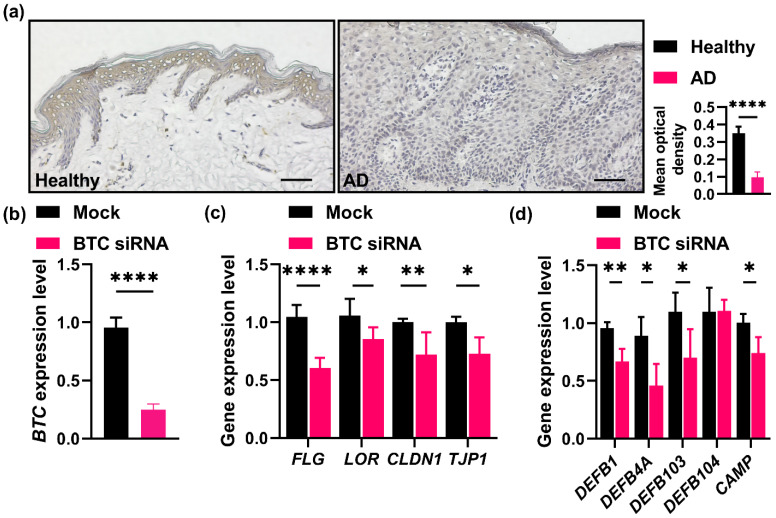
Cutaneous expression and distribution of BTC and effects of its knockdown on human keratinocytes. (**a**). Representative immunohistochemistry images (**left**) and quantification (**right**) of BTC in healthy normal skin tissues and AD lesional skin tissues. Scale bar: 200 μm, *n* = 5. The mRNA expression levels of *BTC* (**b**), *FLG*, *LOR*, *CLDN1*, *TJP1* (**c**), *DEFB1*, *DEFB4A*, *DEFB103*, *DEFB104*, and *CAMP* (**d**) in keratinocytes in the presence or absence of BTC siRNA were detected. *n* = 3. * *p* < 0.05, ** *p* < 0.01, and **** *p* < 0.0001.

Following the examination of the expression and distribution of BTC in the skin biopsy samples of patients with AD and healthy subjects, we observed that BTC was present throughout the entire layer of the epidermis, whereas the highest expression of BTC was detected in the granular layer of healthy subjects (Figure 3a). Interestingly, BTC expression was absent in the lesional skin of the patients with AD, confirming that BTC was downregulated in this skin condition.

To explore the role of BTC in suprabasal keratinocytes, *BTC* gene knockdown was performed by transfecting BTC small interfering RNA (siRNA) into differentiated human keratinocytes following culture in high calcium-containing medium. We confirmed that *BTC* expression was efficiently knocked down (Figure 3b). In these BTC-knockdown keratinocytes, we interestingly observed that the expression of SC barrier-related genes, including filaggrin (*FLG*) and loricrin (*LOR*); TJ barrier-related genes, such as claudin-1 (*CLDN1*) and tight junction protein-1 (*TJP1*); and genes encoding skin-derived antimicrobial peptides, such as *DEFB1*, *DEFB4A*, *DEFB103* and *CAMP* (encoding human β-defensin (hBD)-1, hBD-2, hBD-3 and cathelicidin LL-37, respectively), was remarkably downregulated (Figure 3c,d), which suggests that BTC may play a crucial role in skin epidermal barrier function.

### 2.4. BTC Increases the Expression of TJ Components and Antimicrobial Peptides and Improves the Transepithelial Electrical Resistance (TER) Values in Keratinocytes

**Figure 4:**
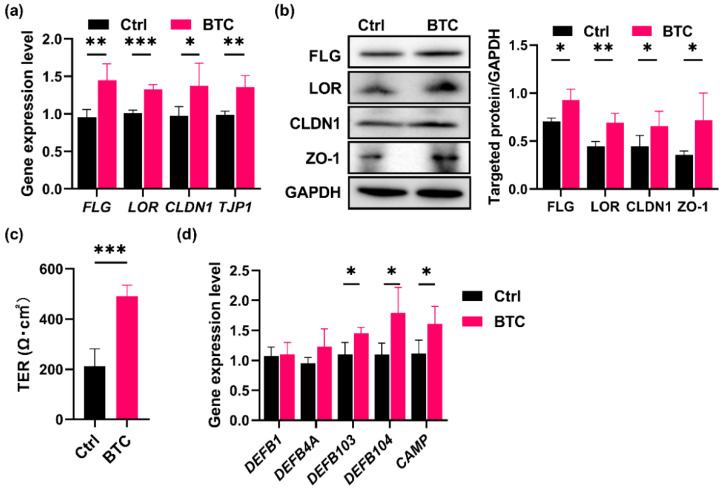
BTC increases the expression of TJ components and skin-derived antimicrobial peptides and improves the TER in keratinocytes. The mRNA expression levels of *FLG*, *LOR*, *CLDN1*, and *TJP1* (**a**); the protein levels of filaggrin, loricrin, claudin-1, ZO-1 (**b**), and TER (**c**); and the mRNA expression levels of *DEFB1*, *DEFB4A*, *DEFB103*, *DEFB104*, and *CAMP* (**d**) in keratinocytes with or without BTC administration were detected. *n* = 3. * *p* < 0.05, ** *p* < 0.01, and *** *p* < 0.001.

To examine the effect of BTC skin barrier components and antimicrobial peptides, differentiated keratinocytes were treated with recombinant human BTC. We found that filaggrin, loricrin, claudin-1, and *zonula occludens*-1 (ZO-1) were simultaneously upregulated at both the mRNA (Figure 4a) and protein levels (Figure 4b). More importantly, the treatment of keratinocyte monolayers with BTC markedly increased the TER, which is the most commonly used parameter to measure the integrity and permeability of a cellular monolayer (Figure 4c), and this finding suggests that BTC improves skin barrier function. Moreover, BTC addition significantly upregulated the gene expression levels of *DEFB103*, *DEFB104*, and *CAMP* (encoding hBD-3, hBD-4, and LL-37, respectively) in keratinocytes (Figure 4d). Taken together, these results indicate that BTC may strengthen the epidermal barrier function and regulate antimicrobial peptide-mediated immunomodulatory functions in keratinocytes.

### 2.5. BTC Recovers the Th2-Type Cytokine-Mediated Impairment of the Skin Barrier in a Skin Equivalent Model

**Figure 5:**
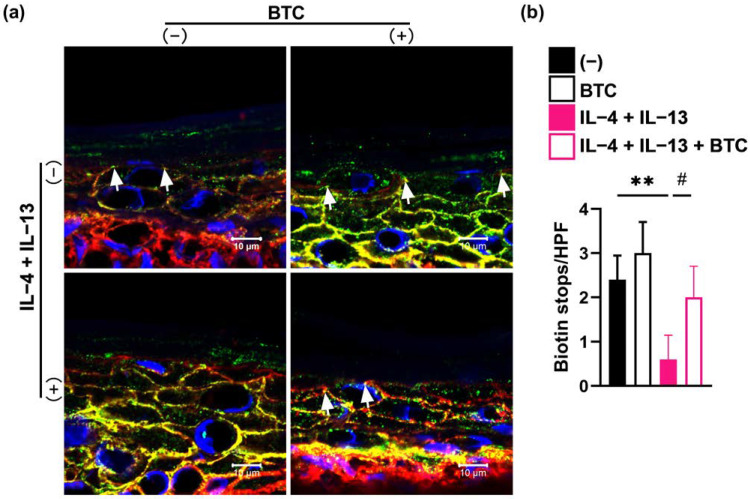
BTC addition leads to recovery of the Th2-type cytokine-mediated impairment of the skin barrier in skin equivalents. Representative immunofluorescence images (**a**) and quantification (**b**) of biotin tracer stops indicated by white arrows in the skin equivalent model, *n* = 4/group. Scale bar: 10 μm. ** *p* < 0.01, and ^#^ *p* < 0.05.

To further evaluate whether BTC may improve the skin barrier function in pathophysiological conditions, we examined the effect of BTC on the skin barrier function in human skin equivalents and in an in vitro AD-like model established by treating skin equivalents with the Th2-type cytokines IL-4 and IL-13 [14]. The epidermal barrier function of skin equivalents was assessed by a tracer penetration assay using an NHS-LC-biotin tracer. We observed that the cytokine cocktail of IL-4 and IL-13 impaired the TJ barrier and increased the permeability of the skin equivalents, leading to easier penetration of the tracer (red color) up to the outermost layer of the epidermis. Notably, following the addition of BTC to IL-4/IL-13 cocktail-treated skin equivalents, tracer penetration was stopped by the TJ barrier, which indicated that BTC restored the IL-4/IL-13 cocktail-induced TJ barrier dysfunction (Figure 5).

### 2.6. BTC Regulates the Skin Barrier Function through EGFR and the Activation of Protein Kinase C (PKC)

**Figure 6:**
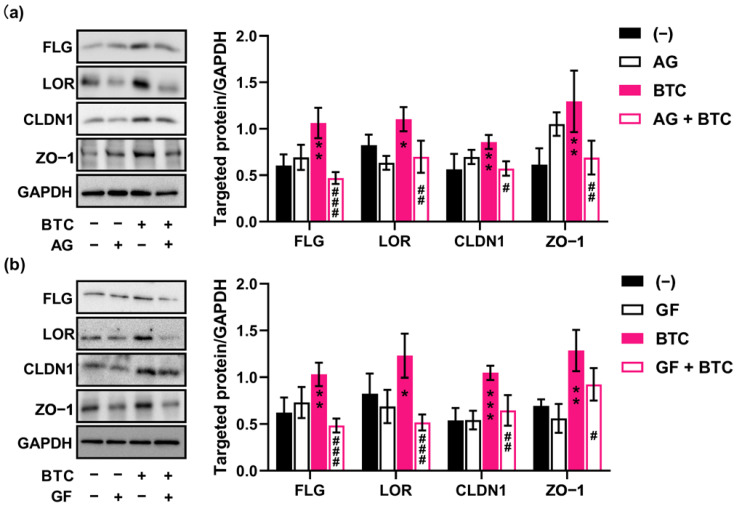
BTC regulates the skin barrier function through the EGFR and PKC signaling pathways. Cells were pretreated with AG-1478 (AG) (**a**) or GF109203X (GF) (**b**) for 2 h and then treated with 100 ng/mL BTC for 24 h. Representative immunoblots of the indicated proteins are shown. The quantification of the band intensities is shown in the right panels, *n* = 4/group. * *p* < 0.05, ** *p* < 0.01, and *** *p* < 0.001 versus the control group; ^#^ *p* < 0.05, ^##^ *p* < 0.01, and ^###^ *p* < 0.001 versus the BTC-treated group.

BTC is one of the ligands of EGFR, a critical receptor that functions to maintain skin homeostasis [3]. To confirm the role of EGFR in BTC-induced skin barrier improvement, keratinocytes were cultured with a selective EGFR inhibitor, AG-1478, prior to treatment with BTC. Notably, BTC failed to upregulate the expression of skin barrier-related proteins such as claudin-1, ZO-1, loricrin, and filaggrin in AG-1478-pretreated keratinocytes (Figure 6a). An interaction between EGFR and PKC pathways in keratinocytes committed to terminal differentiation has been observed [15]. To verify the role of PKC in BTC-regulated skin barrier function, a highly selective, cell-permeable, and reversible PKC inhibitor, GF109203X (GF), was used to inhibit PKC activity in keratinocytes. As shown in Figure 6b, GF remarkably abolished the expression of claudin-1, ZO-1, loricrin and filaggrin in BTC-treated keratinocytes. Collectively, BTC may regulate the skin barrier through the EGFR and PKC signaling pathways.

## 3. Discussion

AD is one of the most prevalent chronic inflammatory skin conditions in the world. Although an increasing number of novel biologicals, such as dupilumab and abrocitinib, have been developed and approved for the treatment of moderate to severe AD [2], there remains a large unmet need in the field of AD management. Therefore, this study aimed to identify novel diagnostic markers and/or therapeutic targets for AD.

In the current study, we first performed a transcriptomic analysis of publicly available datasets, and we observed that *BTC* transcript expression was reduced in AD skin, particularly in skin lesions of patients with AD. Notably, BTC was the most prominently downregulated differentially expressed gene in chronic AD lesional skin (Supplementary Table S1). Although BTC was mainly detected in the upper layers of the epidermis of healthy skin tissues, this molecule was diffusely observed in the full layers of the epidermis. This observation is in line with a previous report showing that BTC expression was restricted to the granular layer of the normal epidermis [8]. Furthermore, the BTC levels were lower in moderate to severe (SCORAD ≥ 25) AD patients. Interestingly, previous studies have demonstrated that BTC expression is reduced in lesional skin of psoriasis, another skin inflammatory condition [9,16,17]. Because BTC exhibits anti-inflammatory properties in inflammatory conditions such as pancreatitis [10,18], it is worth investigating the role of BTC in the pathogenesis of skin inflammatory conditions, including AD.

To explore the role of BTC in differentiated keratinocytes, we examined the phenotype of human keratinocytes following the knockdown and extracellular addition of BTC to keratinocytes. Skin barrier dysfunction is a hallmark of AD pathogenesis, in which the dysregulation of antimicrobial peptides and barrier-related proteins such as filaggrin, loricrin, claudin-1, and ZO-1 (namely, TJP-1) play crucial roles [19]. BTC knockdown resulted in a reduction in antimicrobial peptides and skin barrier-related proteins, whereas BTC addition to keratinocytes enhanced these components, indicating that BTC administration could be a novel therapeutic strategy for AD and other inflammatory skin conditions that are characterized by a dysfunctional skin barrier and dysregulated antimicrobial peptides. In fact, we demonstrated that BTC treatment improved the epidermal barrier function in an in vitro AD-like human skin equivalent model established by treatment with an IL-4 and IL-13 cytokine cocktail. Both IL-4 and IL-13 are the major pathogenic factors in AD lesional skin [1].

BTC is mainly expressed in the stratum granulosum, which is composed of granules containing filaggrin, loricrin, corneodesmosin, and kallikrein, among other compounds [19]. Noticeably, a deficiency of filaggrin or loricrin leads to AD-like skin inflammation and the downregulation of EGFR [13]. Adjacent keratinocytes in the stratum granulosum are connected by TJ proteins, which are mainly classified into the claudin, ZO, and occludin families [19]. Although ZO-1 deficiency delays the formation of the epidermal TJ barrier [20], claudin-1-knockout mice lose the tightness of the TJ barrier and die 24 h after birth [21], which suggests that claudin-1 and ZO-1 play an indispensable role in the formation of the functional epidermal TJ barrier. Importantly, both claudin-1 and ZO-1 are suppressed in AD lesional skin [19]. In the current study, BTC addition enhanced the expression of skin barrier-related proteins and the TER in keratinocytes, and this finding provides insight into the potential therapeutic effect of BTC for AD treatment.

In contrast, antimicrobial peptides, which are also known as host defense peptides, display both antimicrobial and immunomodulatory activities. The antimicrobial peptide-mediated immunomodulatory functions include the promotion of cell proliferation and differentiation, the regulation of cytokine/chemokine production, the acceleration of angiogenesis and wound healing, and the regulation of cell autophagy and the epidermal barrier function [19,22,23]. The downregulation of antimicrobial peptides such as hBD-2, hBD-3, and LL-37 has been observed in lesional skin from AD patients compared with skin tissues from patients with psoriasis [24,25]. Interestingly, both hBD-3 and LL-37 improve the human epidermal keratinocyte barrier function [26,27], and hBD-3 was recently demonstrated to alleviate AD symptoms in an AD-like murine model through autophagy activation [22]. Here, the observation that BTC treatment upregulates both hBD-3 and LL-37 in human epidermal keratinocytes further indicates that BTC may be a novel option for AD treatment. Notably, psoriasis is mediated by T-cell effector cytokines, including IL-17A, IL-22, and TNF-α, which induce antimicrobial peptides in keratinocytes and neutrophils [28,29]. Therefore, although BTC is downregulated in psoriatic skin [9], and BTC knockdown down-regulated antimicrobial peptides, this molecule is most likely not the major inducer of antimicrobial peptides, and its downregulation may be compensated by the overproduced inflammatory cytokines in the lesional skin of psoriasis patients.

BTC is a ligand of EGFR, which plays an important role in skin homeostasis. Here, we found that the BTC-induced enhancement of barrier-related proteins was inhibited by an EGFR inhibitor. Notably, EGF-mediated EGFR signaling has been shown to attenuate *S. aureus*-treated human epidermal keratinocytes and the development and relapse of AD-like skin lesions in NC/Nga mice [12]. Additionally, the PKC signaling pathway was found to be involved in the BTC-mediated activation of keratinocytes. Interestingly, hBD-3 and LL-37 have been shown to improve TJ barrier function through the activation of the PKC pathway [26,27]. Importantly, PKC signaling is controlled by EGFR [30] and regulates keratinocyte differentiation [31,32] and barrier formation [15].

The data presented in this study suggest that the BTC levels are associated with AD severity in skin lesions and that a reduction in the BTC level in the skin of patients with AD may contribute to impaired skin barrier function and the dysregulation of protective antimicrobial peptides. The ability of BTC to improve the skin barrier provides novel insight into the possible biological mechanisms through which BTC may contribute to normal skin physiology and AD pathogenesis. Although additional studies are needed, the potential of BTC to regulate skin barrier function suggests that BTC could become a novel therapeutic target for patients with AD.

## 4. Materials and Methods

### 4.1. Human Subjects

All participants were evaluated based on Hanifin and Rajka’s diagnostic criteria. Biopsy samples of lesional skin were collected from 5 patients with AD, and biopsy samples of healthy skin were obtained from 5 healthy donors. Fresh skin samples were snap-frozen in liquid nitrogen and stored at −80 °C for immunofluorescence analysis. The information of the participants without systemic therapy administration, including investigational agents used for over 4 weeks prior to study entry, was confirmed. Participants with a history of autoimmune diseases, immune deficiency diseases, or tumors were excluded from the study.

### 4.2. Primary Normal Human Epidermal Keratinocytes

Primary normal human epidermal keratinocytes (FC-0007, Kurabo Industries, Osaka, Japan) isolated from neonatal foreskins were cultured in serum-free HuMedia-KG2 keratinocyte growth medium (KK-2150S, Kurabo Industries) containing human epidermal growth factor, insulin, hydrocortisone, gentamicin, amphotericin B, and bovine brain pituitary extract at 37 °C in a humidified atmosphere consisting of 95% air and 5%, as previously described [22]. Elevated concentrations of extracellular Ca^2+^ in cultured keratinocytes induce the formation of TJs and enhance the skin barrier [33]. Therefore, primary human keratinocytes were cultured in 1.8 mM Ca^2+^-containing medium for 24 h to mimic differentiated keratinocytes that form the TJs of the second layer of the stratum granulosum [34]. In addition, keratinocytes were cultured with 100 ng/mL recombinant IL-4 and IL-13 to mimic the features of AD pathology in vitro as reported previously [14]. In some experiments, keratinocytes were treated with inhibitors for 2 h before treatment with BTC.

### 4.3. Transfection of siRNA

The siRNA duplex sense sequence used for *BTC* was 5′-CAAGCAUUACUGCAUCAAtt-3′. The cultured cells were transfected with 30 pmol of the siRNA duplex targeting *BTC* or the scrambled control siRNA (4390843, Invitrogen, Waltham, MA, USA) using Lipofectamine RNAiMAX (Invitrogen) for 48 h according to the manufacturer’s specifications. The transfection efficiency was evaluated by real-time quantitative PCR.

### 4.4. Analysis of Microarray Datasets

All original microarray datasets related to AD were retrieved by searching GEO (https://www.ncbi.nlm.nih.gov/geo, accessed on 26 September 2022) with the keyword “atopic dermatitis”. To retrieve exhaustive, nonredundant data, the following series of criteria were fulfilled: (1) studies with no genome-wide probes or few probes were filtered; (2) for the GSE series with possibly duplicated samples or identical sample resources, the one with a larger sample size was retained, and the other was excluded; (3) only expression profiles of normal skin of healthy subjects and lesional and nonlesional skin of AD were included; and (4) the data retrieval and quality control were double-checked by two investigators. By October 2021, microarray data (GSE59294, GSE32924, GSE36842, GSE27887, GSE58558, GSE95759, GSE99802, GSE107361, GSE120899, GSE130588, GSE133385, GSE133477, and GSE140684) were obtained and included biopsy samples of normal skin from healthy subjects (*n* = 59) and of nonlesional skin (*n* = 298) and lesional skin (*n* = 391) from patients with AD. An overview of the sample compositions of each dataset is shown in Supplementary Table S2, according to Minimum Information About a Microarray Experiment [35]. To ensure data quality, the outliers in the principal component analysis (PCA) of the expression distribution were excluded from this study. Quality plots, including clustering of the data with the grouping setting data normalized to the mean of the normal group, and similarity plots obtained by PCA after exclusion, are provided in Supplementary Figure S1a,b. In addition, normalization and log2 transformation of the dataset were performed using the robust multiarray analysis (RMA) algorithm. A mean-SD-plot of normalized data with group setting and boxplots of normalized and transformed signal log-ratios of all samples are shown in Supplementary Figure S1c,d. To obtain differentially expressed genes, the data were analyzed using the “limma” package of R software after batch effect correction.

### 4.5. Total RNA Extraction and Real-Time Quantitative PCR

Total RNA was extracted from cells using the RNeasy Plus Micro kit (74034, Qiagen, Hilden, Germany) and RNeasy Plus Universal Mini kit (73404, Qiagen), respectively. Reverse transcription of total RNA to first-strand cDNA was performed using ReverTra Ace qPCR RT Master Mix (FSQ-201, Toyobo, Osaka, Japan) according to the manufacturer’s instructions. Real-time PCR was performed using the QuantiTect SYBR Green PCR Kit (204145, Qiagen). The amplification and detection of mRNA were performed using the StepOnePlus Real-Time PCR System (Life Technologies, Carlsbad, CA, USA) following the manufacturer’s specifications. The sequence-specific primer sets used in this study are listed in Supplementary Table S3. All real-time PCRs were performed in triplicate, and the fold changes in gene expression are reported relative to the values found for the untreated controls.

### 4.6. Western Blot Analysis

The samples derived from human keratinocytes were lysed with RIPA lysis buffer (9806, Cell Signaling Technology, Beverly, MA, USA). The protein concentrations were determined using Precision Red Advanced Protein Assay reagent (ADV02, Cytoskeleton, Denver, CO, USA), and equal amounts of total protein were subjected to electrophoresis with 8–15% SDS–PAGE gels followed by transfer to polyvinylidene fluoride (PVDF) membranes (IPVH00010, Merck Minipore, Burlington, MA, USA). The membranes were then blocked in ImmunoBlock buffer for 1 h at room temperature and then incubated overnight at 4 °C with primary antibodies according to the manufacturer’s instructions. The labeling of the primary antibodies was detected using sheep anti-rabbit or sheep anti-mouse antibodies conjugated to horseradish peroxidase (NA934 V and NA931 V, respectively (Amersham Biosciences, Piscataway, NJ, USA)), developed with the Luminata Forte Western horseradish peroxidase substrate (WBLUF0100, Merck Millipore, Billerica, MA, USA), and then imaged using Fujifilm LAS-4000 Plus (Fujifilm, Tokyo, Japan). ImageJ was used for quantification of the band intensities in the images. The antibodies used in these studies are listed in Supplementary Table S4.

### 4.7. Measurements of the TER

The TER measurements were performed as described previously [22]. Briefly, keratinocytes grown on 0.6-cm^2^ Transwell filters were transferred into 1.8 mM Ca^2+^-containing medium, and BTC (100 ng/mL) was added to both the apical and basal compartments. The TER across the keratinocyte monolayers was measured at 48 h poststimulation using cellZscope (NanoAnalytics, Münster, Germany).

### 4.8. Human Skin Equivalent Models

LabCyte EPI-Model24 (J-Tec Co., Aichi, Japan), which consisted of normal human epidermal keratinocytes cultured to form a multilayer, has been shown to provide a highly differentiated model of the human epidermis [36]. Upon arrival, the fully differentiated tissues were aseptically removed from the agarose transport medium, transferred into 24-well plates with assay medium and incubated overnight. On Day 2, the tissues were exposed to BTC in the absence or presence of IL-4 and IL-13 (100 ng/mL) for 72 h. After the treatments, the skin equivalents were fixed with 4% paraformaldehyde in PBS.

### 4.9. TJ Permeability Assay

The TJ permeability assay was performed using EZ-Link^TM^ Sulfo-NHS-LC-Biotin (Thermo Scientific, Waltham, MA, USA) as a paracellular tracer in skin equivalents as previously described [37]. Briefly, skin equivalents were incubated with 2 mg/mL NHS-LC-Biotin in PBS containing 1 mM CaCl_2_ from the dermal side for 30 min. Following incubation, the skin models were immediately embedded in OCT compound and snap-frozen for cryofixation. The frozen sections were subsequently fixed in 4% paraformaldehyde in PBS for 10 min, blocked with ImmunoBlock for 30 min, and then incubated overnight at 4 °C with anti-claudin-1 antibody. After three washes with blocking buffer, the sections were incubated with a mixture of Alexa Fluor 488-conjugated goat anti-mouse antibody and streptavidin Alexa Fluor 594-conjugated antibody for 1 h. After mounting, the images were processed using a Zeiss laser-scanning microscope (LSM) 780 system. Information on all the antibodies used in this study is found in Supplementary Table S2.

### 4.10. Statistical Analyses

All statistical analyses were performed using GraphPad Prism 9 software (GraphPad Software Company, version 9.0.0, San Diego, CA, USA). Student’s t test was used to compare two groups, and one-way analysis of variance (ANOVA) with Tukey’s multiple comparison test was utilized for comparisons of multiple groups. *p* < 0.05 was considered to indicate statistical significance.

## 5. Patents

An application for a patent (No. 2022-095109) named “Testing for a novel atopic dermatitis-associated factor” has been submitted.

## Data Availability

All data generated or analyzed during this study are included in this article.

## Supplementary Materials

The following supporting information can be downloaded: https://www.mdpi.com/article/10.3390/ijms231911520/s1. References [38,39,40,41,42,43,44,45,46,47,48,49,50] are cited in the Supplementary Materials.
