# Supplementary material for: Experimental and Clinical Evidence Suggests That Treatment with Betacellulin Can Alleviate Th2-Type Cytokine-Mediated Impairment of Skin Barrier Function
Source: Int J Mol Sci. 2022 Sep 29;23(19):11520. doi: 10.3390/ijms231911520 (PMC9569883; doi:10.3390/ijms231911520)
Supplement: Supplementary file 1 [file ijms-23-11520-s001.zip › ijms-1859496-supplementary-done.pdf]

International Journal of
Molecular Sciences

MDPI

Supplementary material

Experimental and Clinical Evidence Suggests That Treatment with Betacellulin Can Alleviate Th2-Type Cytokine-Mediated Impairment of Skin Barrier Function

Ge Peng [1,2], Saya Tsukamoto [1,2], Yoshie Umehara [1], Ryoma Kishi [3,4], Mitsutoshi Tominaga [3], Kenji Takamori [3], Ko Okumura [2], Hideoki Ogawa [2], Shigaku Ikeda [1,2] and François Niyonsaba [2,5,*]

1 Department of Dermatology and Allergology, Juntendo University Graduate School of Medicine, Tokyo 1138421, Japan
2 Atopy (Allergy) Research Center, Juntendo University Graduate School of Medicine, Tokyo 1138421, Japan
3 Juntendo Itch Research Center (JIRC), Institute for Environmental and Gender-Specific Medicine, Juntendo University Graduate School of Medicine, Chiba 2790021, Japan
4 Department of Dermatology, Juntendo University Urayasu Hospital, Chiba 2790021, Japan
5 Faculty of International Liberal Arts, Juntendo University, Tokyo 1138421, Japan
* Correspondence: francois@juntendo.ac.jp

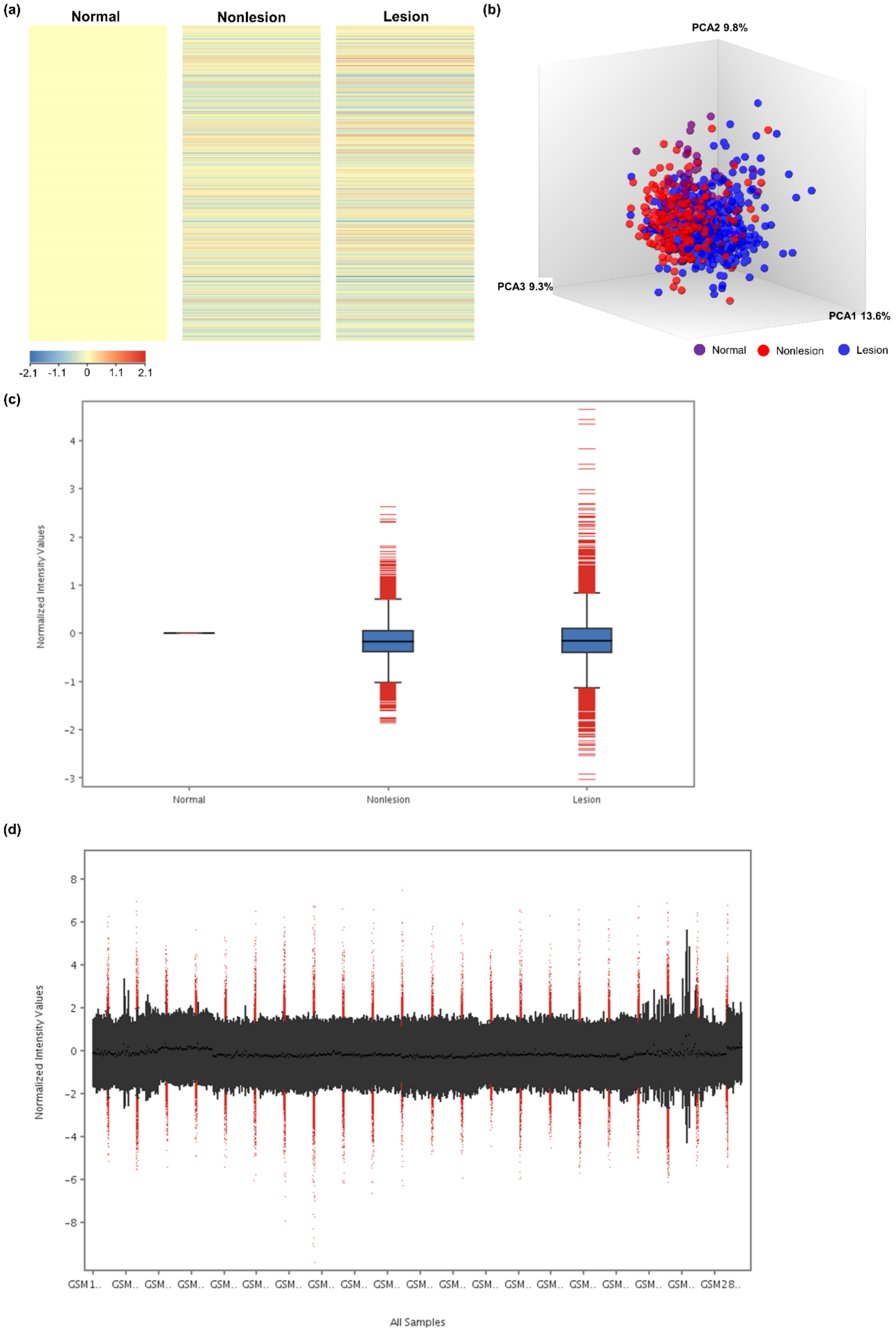

Supplementary Figure S1. Quality plots and comparison plots. (a) Clustering of the data with the grouping setting data normalized to the mean of the normal group. (b) Similarity plots obtained by principal component analysis (PCA). (c) Mean-SD-plot of normalized data with the group setting. (d) Boxplots of normalized and transformed signal log-ratios of all samples.

Supplementary Table S1 Top 10 differential expressed genes identified in this study

Symbol	Description	Fold Change	P-value	FDR P-value
Downregulated				
BTC	betacellulin	-5.17	1.40E-45	1.40E-45
HSD11B1	hydroxysteroid (11-beta) dehydrogenase 1	-3.18	7.31E-40	2.66E-36
RAB3B	RAB3B, member RAS oncogene family	-3.07	3.22E-44	2.92E-40
IL37	interleukin 37	-3	1.22E-27	3.64E-25
WIF1	WNT inhibitory factor 1	-2.69	2.51E-27	6.78E-25
Upregulated				
MMP12	matrix metallopeptidase 12	5.27	2.07E-28	7.19E-26
DEFB4A	defensin, beta 4A	5.44	5.05E-27	1.27E-24
S100A9	S100 calcium-binding protein A9	5.7	1.14E-37	2.49E-34
S100A7A	S100 calcium-binding protein A7A	6.59	2.83E-31	1.70E-28
SERPINB4	serpin peptidase inhibitor, clade B (ovalbumin), member 4	10.78	2.62E-37	5.38E-34

Supplementary Table S2. Characteristics of the microarray datasets included in this study.

GEO accession	Samples					Year	Treatments	Platform	Ref.
	Normal	Nonlesion		Lesion					
		Pre	Post	Pre	Post				
GSE59294	-	7	5	16	12	2014	Dupilumab	GPL570 (HG-U133_Plus_2) Affymetrix Human Genome U133 Plus 2.0 Array	[1]
GSE32924	8	-	-	12	-	2011	-	GPL570 (HG-U133_Plus_2) Affymetrix Human Genome U133 Plus 2.0 Array	[2]
GSE36842	-	8	-	16	-	2012	-	GPL570 (HG-U133_Plus_2) Affymetrix Human Genome U133 Plus 2.0 Array	[3]
GSE27887	-	8	9	9	9	2011	Narrow-band UVB	GPL570 (HG-U133_Plus_2) Affymetrix Human Genome U133 Plus 2.0 Array	[4]
GSE58558	-	20	37	15	27	2014	Cyclosporine	GPL570 (HG-U133_Plus_2) Affymetrix Human Genome U133 Plus 2.0 Array	[5]
GSE95759	-	8	-	14	-	2018	-	GPL570 (HG-U133_Plus_2) Affymetrix Human Genome U133 Plus 2.0 Array	[6]
GSE99802	-	53	88	59	102	2018	Fezakinumab	GPL570 (HG-U133_Plus_2) Affymetrix Human Genome U133 Plus 2.0 Array	[7]

GSE107361	29	40	-	39	-	2018	-	GPL570 (HG-U133_Plus_2) Affymetrix Human Genome U133 Plus 2.0 Array	[8]

(Continued)

GSE120899	2	21	-	20	15	2018	Apremilast	GPL570 (HG-U133_Plus_2) Affymetrix Human Genome U133 Plus 2.0 Array	[9]
GSE130588	20	42	22	51	73	2019	Dupilumab	GPL570 (HG-U133_Plus_2) Affymetrix Human Genome U133 Plus 2.0 Array	[10]
GSE133385	-	30	-	30	51	2019	JAK/SYK-inhibitor ASN002	GPL570 (HG-U133_Plus_2) Affymetrix Human Genome U133 Plus 2.0 Array	[11]
GSE133477	-	40	-	79	121	2019	Crisaborole	GPL570 (HG-U133_Plus_2) Affymetrix Human Genome U133 Plus 2.0 Array	[12]
GSE140684	-	21	24	31	76	2019	Ustekinumab	GPL570 (HG-U133_Plus_2) Affymetrix Human Genome U133 Plus 2.0 Array	[13]
Total	59	298	185	391	486				

Pre: pretreatment; Post: posttreatment

Supplementary Table S3. Primer sequences used for real-time PCR

Primer name	Primer sequence (5'- to -3')
hRPS18	
F	TTTGCGAGTACTCAACACCAACATC
R	GAGCATATCTTCGGCCCACAC
BTC	
F	CCTCTTCGGAAACGTCGTAAA
R	AGCTTGCCACCACCTGGA
CLDN1	
F	GGGCAGATCCAGTGCAAAG
R	GGATGCCAACCACCATCAAG
TJP1	
F	GACCAATAGCTGATGTTGCCAGAG
R	TGCAGGCGAATAATGCCAGA
FLG	
F	GGAATTTCGGCAAATCCTG
R	GCTTGAGCCAACTTGAATACCA
LOR	
F	GGCTGCATCTAGTTCTGCTGTTTA
R	CAAATTTATTGACTGAGGCACTGG

Supplementary Table S4. List of antibodies used in this study

Primary antibodies			
**Antibodies**	**Catalog No.**	**Dilution**	**Company**
Claudin-1	374900	1:100	Invitrogen, Waltham, MA
ZO-1	339100	1:100	Invitrogen, Waltham, MA
Filaggrin	ab24584	1:1000	Abcam, Waltham, MA
Loricrin	ab85679	1:1000	Abcam, Waltham, MA
**Secondary antibodies**			
Sheep anti-rabbit antibody conjugated to HRP	NA934V	1:5000	Cytiva, Marlborough, MA
Sheep anti-mouse antibody conjugated to HRP	NA931V	1:5000	Cytiva, Marlborough, MA

Supplementary References

1. Hamilton, J.D.; Suárez-Fariñas, M.; Dhingra, N.; Cardinale, I.; Li, X.; Kostic, A.; Ming, J.E.; Radin, A.R.; Krueger, J.G.; Graham, N.; et al. Dupilumab improves the molecular signature in skin of patients with moderate-to-severe atopic dermatitis. *J Allergy Clin Immunol* **2014**, *134*, 1293-1300, doi:10.1016/j.jaci.2014.10.013.
2. Suárez-Fariñas, M.; Tintle, S.J.; Shemer, A.; Chiricozzi, A.; Nograles, K.; Cardinale, I.; Duan, S.; Bowcock, A.M.; Krueger, J.G.; Guttman-Yassky, E. Nonlesional atopic dermatitis skin is characterized by broad terminal differentiation defects and variable immune abnormalities. *J Allergy Clin Immunol* **2011**, *127*, 954-964.e951-954, doi:10.1016/j.jaci.2010.12.1124.
3. Gittler, J.K.; Shemer, A.; Suárez-Fariñas, M.; Fuentes-Duculan, J.; Gulewicz, K.J.; Wang, C.Q.; Mitsui, H.; Cardinale, I.; de Guzman Strong, C.; Krueger, J.G.; et al. Progressive activation of T(H)2/T(H)22 cytokines and selective epidermal proteins characterizes acute and chronic atopic dermatitis. *J Allergy Clin Immunol* **2012**, *130*, 1344-1354, doi:10.1016/j.jaci.2012.07.012.
4. Tintle, S.; Shemer, A.; Suárez-Fariñas, M.; Fujita, H.; Gilleaudeau, P.; Sullivan-Whalen, M.; Johnson-Huang, L.; Chiricozzi, A.; Cardinale, I.; Duan, S.; et al. Reversal of atopic dermatitis with narrow-band UVB phototherapy and biomarkers for therapeutic response. *J Allergy Clin Immunol* **2011**, *128*, 583-593.e581-584, doi:10.1016/j.jaci.2011.05.042.
5. Khattri, S.; Shemer, A.; Rozenblit, M.; Dhingra, N.; Czarnowicki, T.; Finney, R.; Gilleaudeau, P.; Sullivan-Whalen, M.; Zheng, X.; Xu, H.; et al. Cyclosporine in patients with atopic dermatitis modulates activated inflammatory pathways and reverses epidermal pathology. *J Allergy Clin Immunol* **2014**, *133*, 1626-1634, doi:10.1016/j.jaci.2014.03.003.

6. Malik, K.; Ungar, B.; Garcet, S.; Dutt, R.; Dickstein, D.; Zheng, X.; Xu, H.; Estrada, Y.D.; Suárez-Fariñas, M.; Shemer, A.; et al. Dust mite induces multiple polar T cell axes in human skin. *Clin Exp Allergy* **2017**, *47*, 1648-1660, doi:10.1111/cea.13040.
7. Brunner, P.M.; Pavel, A.B.; Khattri, S.; Leonard, A.; Malik, K.; Rose, S.; Jim On, S.; Vekaria, A.S.; Traidl-Hoffmann, C.; Singer, G.K.; et al. Baseline IL-22 expression in patients with atopic dermatitis stratifies tissue responses to fezakinumab. *J Allergy Clin Immunol* **2019**, *143*, 142-154, doi:10.1016/j.jaci.2018.07.028.
8. Brunner, P.M.; Israel, A.; Zhang, N.; Leonard, A.; Wen, H.C.; Huynh, T.; Tran, G.; Lyon, S.; Rodriguez, G.; Immaneni, S.; et al. Early-onset pediatric atopic dermatitis is characterized by T(H)2/T(H)17/T(H)22-centered inflammation and lipid alterations. *J Allergy Clin Immunol* **2018**, *141*, 2094-2106, doi:10.1016/j.jaci.2018.02.040.
9. Simpson, E.L.; Imafuku, S.; Poulin, Y.; Ungar, B.; Zhou, L.; Malik, K.; Wen, H.C.; Xu, H.; Estrada, Y.D.; Peng, X.; et al. A Phase 2 Randomized Trial of Apremilast in Patients with Atopic Dermatitis. *J Invest Dermatol* **2019**, *139*, 1063-1072, doi:10.1016/j.jid.2018.10.043.
10. Guttman-Yassky, E.; Bissonnette, R.; Ungar, B.; Suárez-Fariñas, M.; Ardeleanu, M.; Esaki, H.; Suprun, M.; Estrada, Y.; Xu, H.; Peng, X.; et al. Dupilumab progressively improves systemic and cutaneous abnormalities in patients with atopic dermatitis. *J Allergy Clin Immunol* **2019**, *143*, 155-172, doi:10.1016/j.jaci.2018.08.022.
11. Pavel, A.B.; Song, T.; Kim, H.J.; Del Duca, E.; Krueger, J.G.; Dubin, C.; Peng, X.; Xu, H.; Zhang, N.; Estrada, Y.D.; et al. Oral Janus kinase/SYK inhibition (ASN002) suppresses inflammation and improves epidermal barrier markers in patients with atopic dermatitis. *J Allergy Clin Immunol* **2019**, *144*, 1011-1024, doi:10.1016/j.jaci.2019.07.013.
12. Bissonnette, R.; Pavel, A.B.; Diaz, A.; Werth, J.L.; Zang, C.; Vranic, I.; Purohit, V.S.; Zielinski, M.A.; Vlahos, B.; Estrada, Y.D.; et al. Crisaborole and atopic dermatitis skin biomarkers: An intrapatient randomized trial. *J Allergy Clin Immunol* **2019**, *144*, 1274-1289, doi:10.1016/j.jaci.2019.06.047.
13. Khattri, S.; Brunner, P.M.; Garcet, S.; Finney, R.; Cohen, S.R.; Oliva, M.; Dutt, R.; Fuentes-Duculan, J.; Zheng, X.; Li, X.; et al. Efficacy and safety of ustekinumab treatment in adults with moderate-to-severe atopic dermatitis. *Exp Dermatol* **2017**, *26*, 28-35, doi:10.1111/exd.13112.
